# Prevalence, Knowledge, and Attitudes among Health Professions Students toward the Use of Electronic Cigarettes

**DOI:** 10.3390/healthcare10122420

**Published:** 2022-11-30

**Authors:** Suha AlMuhaissen, Haneen Mohammad, Afnan Dabobash, Marya Q. Nada, Zahra M. Suleiman

**Affiliations:** 1 Department of Pharmaceutics and Pharmaceutical Technology, School of Pharmacy, University of Jordan, Amman 11942, Jordan; 2 Department of Pharmaceutical Sciences, School of Pharmacy, University of Jordan, Amman 11942, Jordan

**Keywords:** electronic cigarettes, smoking, nicotine, prevalence, attitude, knowledge, health professions students

## Abstract

Healthcare professionals are standing against the widespread use of e-cigarettes among the population, especially young adults. E-cigarettes are generally introduced, especially via social media platforms, as a safer alternative to conventional cigarette smoking. The aim of this study was to measure the prevalence of e-cigarette use among healthcare professions students at the University of Jordan, as well as to explore their knowledge and attitudes toward the use of e-cigarettes. An anonymous Google-Form^®^-based cross-sectional questionnaire was presented to potential participants at the University of Jordan. Students’ responses were analyzed using SPSS^®^ 23.0 software. A total of 679 online surveys were completed. About 37.4% of students reported using e-cigarettes at least once in their lifetime and about 20% of students were current e-cigarette users. The multivariate analysis showed that the factors associated with E-cigarette smoking are being male, having mothers, siblings, or friends using e-cigarettes, having easy access to e-cigarettes, and having social media as the main source of knowledge regarding e-cigarettes. The relatively high acceptance level and use of e-cigarettes among health professions students should be an alarming sign to decision-makers to give more attention to legislation that controls tobacco products including e-cigarette selling and advertising.

## 1. Introduction

Electronic cigarettes (e-cigarettes) are battery-powered electronic devices that aerosolize liquids into inhalable vapors. The vaporized liquids may contain nicotine, solvents, and a wide range of flavoring agents [1]. E-cigarettes were invented initially as smoking cessation aids, such as more conventional types of nicotine replacement therapies, such as nicotine patches or gums. E-cigarettes that include nicotine may help to reduce cravings and withdrawal symptoms by replacing the nicotine found in tobacco smoke. Moreover, e-cigarettes may help smokers quit by resolving the sensory and behavioral aspects of tobacco addiction [2]. However, the use of e-cigarettes could be associated with some harmful health consequences including the addictive effect of nicotine consumption, the toxicants and heavy metals delivered through the vapor to the user, and the risk of battery explosion [3].

Although the e-cigarettes effectiveness and safety of e-cigarettes for quitting smoking are still controversial, in recent years, their use has increased dramatically, especially among teenagers and young people [4,5]. In 2018, 8.1 million adults in the United States smoked e-cigarettes, with the age group 18 to 24 having the highest prevalence according to the Centers for Disease Control and Prevention (CDC) [6]. Moreover, there was a marked increase in the daily usage of e-cigarettes from 2018 to 2020 among young US adults aged 21 and 24, with a prevalence of 4.4% to 6.6%, respectively [7].

At a Saudi university, 27.7% of health science students use e-cigarettes, which is roughly double the rate of students who smoke conventional cigarettes [8]. A national survey conducted in New Zealand in 2018 showed that 40.5% of university students had ever used e-cigarettes [9]. In China, surveys that have been conducted between 2009 and 2014 showed an increase in the rate of smokers who had ever used e-cigarettes from 2% to 11%, respectively. Additionally, compared to other age groups, people aged between 15 and 24 were more likely to have tried e-cigarettes (4.1%) [10].

In Jordan, the situation is not ideal regarding university students’ knowledge about and attitudes toward using e-cigarettes. According to a previous study, 60% of university students in Jordan believed that all e-cigarettes contained natural ingredients, 40% of the students agreed that using e-cigarettes is less addictive than using conventional cigarettes, and 30% of them believed that using e-cigarettes is less risky than using conventional cigarettes [11].

Given the sharp increase in e-cigarette use among university students, it is urgent to understand the parameters related to their usage. Several studies have shown that users have a variety of justifications for using e-cigarettes, including low cost, availability, ease of use, and attractive flavorings and smells, as well as curiosity, quitting smoking, health benefits, and use by a friend or a family member. Additionally, it was indicated that incorrect knowledge may encourage more university students to use e-cigarettes [12,13].

Al-Balas and colleagues reported different sources of knowledge about e-cigarettes among different age groups within the Jordanian population. Friends were most frequently mentioned (72.2%), followed by the Internet (23%), vape shops (1.9%), TV (1.8%), and neighborhood (1%) [14].

According to the Jordanian Food and Drug Administration (JFDA) legislation of 2019 that regulates e-cigarette use, the advertisement and promotion of e-cigarette devices and e-liquids in any public place are prohibited, and JFDA approval is required for any display inside the sales location. Moreover, advertising for e-cigarettes is not allowed anywhere, even online or on social media [15]. That makes the internet and vending machine sales of e-cigarettes forbidden. Additionally, Jordanian Public Health Law No. 47 of 2008 does not allow the use of e-cigarettes in smoke-free areas. Furthermore, e-cigarettes must not be sold to people less than 19 years of age.

In light of the fact that there is inadequate research regarding Jordanian university students’ attitudes toward and knowledge about the hazardous effects of e-cigarettes, more research is required to thoroughly assess their knowledge about and attitudes toward the use of e-cigarettes, especially for health professions students. Healthcare providers play a vital role in improving the access and quality of healthcare for the population. They offer fundamental health services that support wellness, prevent disease, and provide primary healthcare to individuals, families, and communities [16]. Health professions students are the future healthcare providers; therefore, it is important to assess their perception and knowledge about e-cigarettes, which include all varieties of nicotine-containing e-cigarettes sold in Jordan (disposable e-cigarettes and refillable tanks that can be loaded with juice/nicotine salt). Accordingly, the current study aimed (1) to determine the prevalence of e-cigarette smoking among the health professions students at the University of Jordan and (2) to assess possible correlates of e-cigarette usage, such as sociodemographic characteristics, knowledge about, and attitudes toward e-cigarettes.

## 2. Materials and Methods

### 2.1. Data Collection Instrument

A cross-sectional questionnaire was developed based on an intensive review of the available literature [11,13,17,18]. The tool was constructed to study the prevalence of e-cigarette use as well as the knowledge about and attitude toward e-cigarettes among the students from health professions at the University of Jordan (UJ). There are five health professions schools on the campus of the UJ. These schools (with the total number of students in each) are the School of Medicine (3029 students), School of Dentistry (2033 students), School of Pharmacy (2240 students), School of Rehabilitation (1374 students), and School of Nursing (1228). The questionnaire was designed into four sections. The first section constituted the items related to demographic data and sample characteristics including age, gender, nationality, monthly allowance, school, academic level, and GPA. The second section aimed to study the exposure of students to e-cigarettes through questions about whether they have ever used them and if yes, the frequency and duration of use. Moreover, section two included questions about the experience of first-degree relatives of study participants with e-cigarettes. The third section measured study participants’ knowledge about e-cigarettes. In this section, five items were used to investigate knowledge about the components, source, and carcinogenicity of e-cigarettes, whether e-cigarettes can cause addiction or not, and whether e-cigarettes are approved by the FDA to aid in smoking cessation or not. For the purpose of knowledge score calculations, the correct answer was coded as 1, and the incorrect or “I don’t know” answers were coded as 0 as both answers indicate a lack of knowledge. The last section was composed of items concerning the attitude of study participants toward e-cigarettes using the scale that was developed and validated by Fang et al. in 2022 [13]. The original scale was published in English and consists of 19 items with a five-point Likert scale response that ranges from strongly disagree to strongly agree. The scale is divided into five domains: (1) Accessibility, measured using two items which are the ease of access to e-cigarettes in terms of price and storage, (2) Acceptability, evaluated via two items to cover the level of e-cigarettes’ social acceptance, (3) Safety, investigated using four items considering how safe e-cigarettes are for health, (4) Supervision, where four items were used to evaluate whether or not it is important to restrict minors’ access to e-cigarettes, and finally, (5) Restriction. In the original scale, three items were utilized to evaluate if online promotion and selling of e-cigarettes should be banned or not. In the current study, all items were adapted except for the restriction domain, in which only the first three items from the original scale were used.

### 2.2. Attitude Scale Translation

The scale was first translated forward to Arabic by two of the authors. The second step was the evaluation of the translated version by all authors, followed by a backward translation into English which was done by two external bilingual individuals, one of which is a student from a health professions school. Modifications were done and the forward-backward translation steps were done till no further differences were detected. 

The final questionnaire version (Arabic) was evaluated using piloting and statistical evaluation. A pilot study was carried out on the study population (students from health professions at the University of Jordan) and 40 responses were collected. The results from the pilot study were used to modify the tool; mainly, a rearrangement of the questionnaire was suggested and done. Further statistical evaluation of the used scale reliability, test-retest consistency, and sample adequacy were conducted. Cronbach’s alpha value of 0.763 indicated an adequate internal consistency of the tool. The intra-class correlation coefficient value of 0.719 confirmed test-retest reliability. Finally, sample adequacy was assured using Principal Components Analysis (PCA) with Kaiser-Meyer-Olkin (KMO) equal to 0.784 and a significant Bartlett’s Test (*p* = 0.000).

### 2.3. Data Collection

Data collection was carried out by two of the authors during August 2022 over three weeks including weekends. The study questionnaire was administered in simple Arabic via electronic version using Google^®^ Forms. For this purpose, a QR code was created. The data collectors approached students from the five health professions schools located at the University of Jordan and asked them to scan the QR code and fill out the form. The QR code was made available on the announcement board for each school. In addition, the QR code as well as the questionnaire link was distributed through student groups on social media (Facebook^®^). The QR code and the link were published with the following message: “Hello, we are a group of researchers from the School of Pharmacy, at the University of Jordan. We are conducting research on e-cigarette use, knowledge, and attitude toward e-cigarettes among the students in the health professions community at UJ. Because you are a future health care provider and your input is extremely valuable, we would appreciate it if you could fill in the attached questionnaire on the topic of e-cigarettes. You don’t need to write your name or any identifiers and it will take around 5 min to fill it in. Your response will be confidential and only used for scientific research purposes. While filling in the questionnaire, please keep in mind that the term e-cigarette used in our study is intended to include the varieties of e-cigarettes sold in Jordan (disposable e-cigarettes and refillable tanks that can be loaded with juice/nicotine salt). Thank you.” Also, at the beginning of the form, a short summary of the study and its aim as well as information about the estimated time required to fill the form were given. Informed consent was obtained to fulfill the questions/survey. 

### 2.4. Ethical Approval

The study was approved by the Ethical Committee of the Deanship of Academic Research at the University of Jordan (decision number 91-2022). To minimize social desirability bias, participants were assured that data were collected in accordance with the code of conduct of research with human subjects in Jordan, and they were coded and kept anonymously and confidentially.

### 2.5. Data Analysis

Sample size calculation was done based on e-cigarette use prevalence values (P) reported in the literature [17], with Zα = 1.96 and d = 0.05. Using the values of reported prevalence, the calculation illustrated that a maximum of 340 participants were enough to ensure a reliable sample size to study e-cigarette prevalence and attitudes toward e-cigarettes among health professions students at the University of Jordan. To minimize sample bias, oversampling was insured (679 responses instead of 340 responses). The online questionnaire QR code was distributed through all social media groups of the five health professions schools and hung on announcement boards in all these schools, maintaining a convenient questionnaire time and proper question flow.

Data analysis was carried out using SPSS^®^ 23.0 (IBM, Chicago, IL, USA). Continuous variables were presented as mean and standard deviation (or median and interquartile ranges) while categorical variables were presented as count and percentages. Chi-square or the independent sample Mann-Whitney-U tests were used to detect differences between different groups. Logistic regression was conducted to study factors that affect the dichotomous variable of being an ever e-cigarette user (Yes/No) as a dependent variable. Factors that showed significant differences between groups with ever e-cigarette users upon single testing were included except for the conventional cigarette use covariate as this factor yielded a high odds ratio and wide confidence interval upon initial screening. All hypothesis testing was two-sided. A *p*-value of <0.05 was considered significant.

## 3. Results

### 3.1. Participants’ Characteristics

Table 1 shows the study sample characteristics including demographic and distribution of e-cigarette ever users and non-users. A total of 679 students in health professions schools at the University of Jordan responded to the questionnaire, with 77.5% being female and the mean age of participants being 20.5 ± 2.1 years. The vast majority are Jordanians (92.6%). The participants from the five health professions schools were distributed as follows: 15.9% from the School of Medicine, 20.6%from the School of Dentistry, 22.1% from the School of Pharmacy, 24.6% from the School of Rehabilitation Sciences, and 16.8% from the School of Nursing.

About three-quarters of the participants (73.5%) live in Amman. Regarding the living conditions, the majority of participants (89.4%) live with their families. Study participants are from all academic levels, mainly (47.4%) with very good GPA levels. 

As also shown in Table 1, more than one-third (252, 37.1%) of the study sample have ever used e-cigarettes. The sample characteristics that are significantly associated with being a user of e-cigarettes include male gender (χ^2^ = 44.8, *p* < 0.001), students from the School of Rehabilitation Sciences and the School of Dentistry (χ^2^ = 24.3, *p* < 0.001), first, second, and third year academic levels (χ^2^ = 8.7, *p* = 0.019), low GPA categories (χ^2^ = 17.5, *p* = 0.002), living with friends or alone (χ^2^ = 8.5, *p* = 0.014), and monthly allowance higher than $140 (χ^2^ = 15.7, *p* < 0.001).

### 3.2. Participants’ Exposure to E-Cigarettes

Table 2 demonstrates the results of participants’ exposure to e-cigarettes. More than two-thirds of the fathers and mothers of the students received a diploma or higher education (70.7% and 70.8%, respectively). There is a significant statistical association between the level of fathers’ education and the chance of the participants using e-cigarettes (*p* = 0.045). Participants whose fathers’ education was a diploma or more have a higher tendency to use e-cigarettes.

Of all respondents, 39 (5.7%) reported current conventional cigarette use, of which 38 (97.4%) are e-cigarette ever-users and 1 (2.6%) is an e-cigarette non-user. Of the study participants, 21 (3.1%) are conventional cigarette ex-smokers, with 20 (95.2%) having ever used e-cigarettes. Noticeably, there is a statistically significant association between being an ex-smoker or a current user of conventional cigarettes and using e-cigarettes (χ^2^ = 100, *p* < 0.001). Additionally, 199 (29.3%) of study participants stated that their fathers had ever used e-cigarettes while 78 (11.5%) of study participants confirmed that their mothers had at least tried e-cigarettes. Almost half of the study participants’ siblings and more than two-thirds of their friends had ever used e-cigarettes. As expected, having a father, a mother, a sibling, or a friend who had ever used e-cigarettes is significantly associated with being an e-cigarette user.

### 3.3. E-Cigarette Use and Its Correlates

Table 3 demonstrates the frequency, duration of E-cigarette use, and source of information about e-cigarettes. The percentage of respondents who used e-cigarettes in the past and just tried them is 17.4%, leaving around 19.7% as current users (8.8% have been using e-cigarettes for 1 year, 8.8% have been using e-cigarettes for 1 to 3 years, and 2% started using e-cigarettes more than 3 years ago). On the other hand, 28.6% of current e-cigarette users do use them on a daily basis while 9.1% and 15.5% of respondents use e-cigarettes on a weekly and monthly basis, respectively.

The main two sources of information about e-cigarettes are social media (80.9%), followed closely by friends (79.7%), followed by university lectures and seminars (59%). Surprisingly, tobacco shops were listed by only 1.2% of respondents as a possible source of information about e-cigarettes.

### 3.4. Knowledge about E-Cigarettes

As shown in Table 4, approximately three-quarters of the participants considered that e-cigarettes are carcinogenic (72.3%) and that e-cigarettes contain nicotine (72.3%). Additionally, more than two-thirds of them (70.7%) identified e-cigarettes as tobacco products, and 51.5% of participants were sure that e-cigarettes are addictive. However, only 10.8% of the health professions students were sure that e-cigarettes are FDA-approved as a smoking cessation aid. The overall mean knowledge score for study participants is 2.8 ± 1.1. Interestingly, e-cigarettes ever users are more knowledgeable about e-cigarettes as a significantly higher percentage (*p* < 0.001) of e-cigarette ever users knew that e-cigarettes are carcinogenic (82.9%) and are not an FDA-approved product (16.7%). Moreover, e-cigarette ever users have a significantly higher overall knowledge score (*p* = 0.016; 2.9 ± 1).

### 3.5. Attitudes towards E-Cigarettes

Table 5 elucidates the study participants’ attitudes toward e-cigarettes as classified by five dimensions. E-cigarette ever users exhibit a significantly higher positive attitude toward e-cigarettes in four out of five dimensions. These are acceptability (*p* = 0.030), safety (*p* < 0.001), supervision (*p* < 0.001), and restriction (*p* < 0.001).

### 3.6. Covariates Connected to E-Cigarette Use

Table 6 shows the factors affecting the tendency to be an e-cigarette ever user. Results of the binary logistic regression showed that male gender, mother, siblings, and friends being E-cigarette users, media, accessibility, and safety have effects significantly different from zero on the possibility of being an e-cigarette ever user.

## 4. Discussion

The community sees healthcare professionals as the source of the most trusted knowledge regarding health. Hence, healthcare professionals play a vital role affecting the smoking rate in the community by providing the right advice to e-cigarette users [19]. For instance, a study has shown that some young people would reduce or quit smoking if they were informed by their dentist regarding the harmful effects of smoking on their oral health [20]. Consequently, health professions students need to be well educated about e-cigarettes as they may encounter e-cigarette users when they start their careers as healthcare professionals, so they can make the best patient counseling and clinical judgments.

The results of the current study have shown a high level of knowledge regarding e-cigarettes among health professions students. More than 70% of the participants were aware that e-cigarettes contain nicotine, are considered tobacco products, and can cause cancer. The level of knowledge that students have in this study is higher than that reported at Hangzhou University in China, where only around 58% of students were sure that e-cigarettes contain nicotine and more than 68% of students didn’t identify e-cigarettes as tobacco products [13]. 

However, about 48.5% of the participants were not aware that e-cigarettes are addictive. In fact, the addictiveness to nicotine in e-cigarettes is considered one of the major health concerns regarding the use of e-cigarettes, which could lead to lifelong e-cigarettes usage, especially in the youth population. Studies have shown that the serum nicotine level with e-cigarette use can be close to the serum nicotine level with conventional cigarette use [21].

Almost 90% of participants were not sure whether e-cigarettes are FDA-approved as smoking cessation aids or not. In fact, to date of this publication, e-cigarettes products have not been approved by the FDA as smoking cessation aids [22]. Those findings are comparable to the results reported at a Saudi university where the majority of medical students didn’t know that e-cigarettes were not approved by the FDA as a smoking cessation aid [18]. Moreover, a meta-analysis of 26 studies has shown that the probability of adult smokers quitting smoking is lower in smokers who use e-cigarettes for smoking cessation compared to non- e-cigarette users [21].

In this study around 20% of students were current e-cigarette smokers; this percentage is lower than the prevalence of e-cigarette smoking reported among Jordanian adults (33.1%) [17]. This may be justified by the high level of knowledge of health professions students which makes them more aware of the harmful effects of e-cigarettes. On the other hand, the prevalence in this study was higher than that reported among Jordanian university students which was only around 10% as measured by a national cross-sectional-questionnaire-based study. The respondents to the mentioned study were 1259 undergraduate and graduate Jordanian university students, of which 63.7% were enrolled in medical schools, and similar to our study, most of the mentioned study participants were females (70.2%) [11]. Furthermore, a national cross-sectional questionnaire-based study from Jordan, which included 1819 medical students from five Jordanian medical colleges, showed that the prevalence of e-cigarette use among Jordanian university medical students was 9.5% e-cigarettes [23]. Compared to the prevalent results reported in the previously cited studies and taking into consideration that the methods used in those studies may not be similar, the prevalence reported in this study indicates an increase in the e-smoking rate among university students in Jordan which could be an alarming sign of the increase of e-cigarettes smoking popularity among young adults in Jordan. Looking at neighboring countries, a study from Saudi Arabia has shown that the prevalence of e-cigarette smoking was more at 11.5% among medical students [18].

Regarding the co-factors associated with students being e-cigarette users, our study shows that male students use e-cigarettes about two times (2.3) more than female students. In fact, nearly eight out of ten males in Jordan smoke nicotine products, including e-cigarettes, according to a study carried out by the Jordanian Ministry of Health and the World Health Organization (WHO) [24]. Another study has shown that half of Jordanian males (50.1%) have smoked different tobacco products while only 15.9% of females have. Hence, nearly two-thirds of males in Jordan are current smokers of different tobacco products. That is, males in Jordan have a higher probability to smoke, compared to females. This can be attributed to sociocultural approval which accepts male smoking more than female smoking [25].

Our results show that having a close relationship with e-cigarette smokers increases the probability of students trying or using e-cigarettes. Parents in general have a very high impact on their children’s lives. Hence, students who have e-cigarette smoking mothers are two-fold more susceptible to trying e-cigarettes than students with non-smoking mothers. Similar findings were observed in a meta-analysis of the risk of smoking for adolescents [26]. In addition, having e-cigarette smoker friends or siblings increases the probability of students being an e-cigarette smoker by more than three and four times, respectively. This emphasizes how the social interactions of human beings—especially young adults—greatly affect their behaviors and thoughts in what is known as the chameleon effect [27]. Those findings are consistent with the results of studies conducted in China, Saudi Arabia, and Qatar, which demonstrate the high effect of e-cigarette smokers who have a close relationship, such as a sibling or a friend, on a person’s probability of using e-cigarettes [13,18,28].

Nowadays, social media platforms play an essential role in student life as they are used on a daily basis. This was reflected in the results of our study, where social media platforms were the main source of information regarding e-cigarettes for the majority of students (80.9%), and they have the largest effect on students compared to other sources. Large exposure to e-cigarette advertising posts on social media platforms can motivate young adults to attempt e-cigarette use, as seen in a study among college students in Hawaii, which makes it essential to have good monitoring of advertising posts on all social media platforms [29]. 

In our study, a positive correlation was found between students’ GPAs and their probability to be e-cigarette smokers. Students with fair and weak GPAs were more likely to use e-cigarettes compared to students with higher GPAs, which goes in line with a study conducted on adolescents [30]. Robert and colleagues have shown that the prevalence of smoking is higher among adolescents who have low academic performance [30]. Another study conducted at Cardiff University has shown that non-smoking students have a higher academic performance compared to smoking students, which could be attributed to the negative effects of tobacco smoke on the brain. In addition, students’ performance could be negatively affected by the withdrawal effects of nicotine during assessment periods [31]. 

It was also noticed in the results of our study that when the educational level of the participant’s father increases, the probability of the student being an e-cigarette user increases. This could be explained by the increase in the family income as the father’s educational level increases, so students could have a higher allowance making e-cigarettes more affordable to them. Hence, in our study students with a higher monthly allowance have more chance to be e-cigarette users compared to students with a lower monthly allowance. 

The outcomes of our study have shown an association between conventional cigarette use and e-cigarette use as around 97% of conventional cigarette smokers were e-cigarette users as well. In addition, almost 95% of conventional cigarette ex-smokers had ever used e-cigarettes. In fact, substantial evidence from several studies has shown that smoking e-cigarettes is linked to subsequent conventional cigarette initiation. A study has shown that the likelihood to be a conventional cigarette user is five times more among current e-cigarette smokers [32]. 

E-cigarette smoking is highly perceived to be less harmful than conventional cigarette smoking among young adults. In the present study, most students believe that e-cigarettes are a safer alternative to conventional cigarettes. That was also found in a similar study among university students in Jordan, where most students think that e-cigarette smoking is safer than conventional cigarette smoking [11]. In fact, e-cigarettes vapor is considered very harmful and is linked to oral squamous cell carcinoma [33]. Moreover, the nicotine contained in e-cigarettes is not only addictive, but can also lead to mouth cancer [34]. Furthermore, nicotine has been linked to serious problems affecting the parts of the brain responsible for impulse control, attention, mood, and learning especially in adolescent-developing brains, and increases the risk of future addiction to other drugs. Actually, brains keep developing till the age of 25, making adolescents and young adults highly affected by the negative outcomes of e-cigarettes [35]. In addition, e-cigarette smoking was also associated with lifetime eating disorders such as anorexia nervosa, binge eating, or bulimia as reported by a study among college students in the United States [36]. All the mentioned harmful effects of e-cigarettes necessitate the supervision and control of e-cigarette use.

A study has shown that applying taxes, banning sales and advertising of e-cigarettes designs and their liquid (juice), and increasing awareness about their risks can be helpful in restricting the use of e-cigarettes [37]. A study established in Jordan and the West Bank of Palestine has demonstrated the importance of taxes in reducing the prevalence of tobacco products and the economic burden associated with it. The fact that people usually substitute different tobacco products such as conventional cigarettes, water-pipes, and e-cigarettes shows the importance of applying taxes on all tobacco products, not only on certain products [25].

In our study, most students think that e-cigarettes are harder to obtain and more expensive compared to conventional cigarettes. Hence, the accessibility dimension was the least supported by participants. In fact, e-cigarettes in Jordan are sold mainly in special vape shops while conventional cigarettes can be found in most supermarkets and local shops which makes them easier to purchase. That confirms the importance of restricting the sale of e-cigarette products to special vape shops only, hindering the easy access of youth and young adults to those products.

### Study Limitations

Our study has some limitations. The participants surveyed were students at the University of Jordan only. Therefore, generalizing those results to other student communities of health professions could be limited. Future work including more universities and larger sample sizes is needed to have a more comprehensive understanding of e-cigarette use among university students in Jordan. In addition, the ability to ensure data quality via online questionnaires is lower than in direct interviews. The study population is mainly composed of female participants which might affect the overall incidence rate of e-cigarette use, yet the revealed e-cigarette ratio is considered relatively high. Moreover, based on the registration unit at UJ records at the time of the study, the percentage of female students at the health professions schools is almost two-thirds (65.8%) 

## 5. Conclusions

Our investigations show that the health professions students at the University of Jordan have high knowledge regarding e-cigarettes. Nevertheless, a relatively high prevalence of e-cigarette use was revealed, which is higher than that reported in previous studies in Jordan. This indicates an increase in the popularity and usage of e-cigarettes among university students. In addition, the factors that were associated with students having a higher probability of using e-cigarettes if they are male, have mothers, siblings, or friends using e-cigarettes, have easy access to e-cigarettes, or have social media as their source of knowledge about e-cigarettes. The government and different authorities are advised to pay attention to the present study together with the results from previous studies as they represent an alarming sign of the widespread use of e-cigarettes and insufficient knowledge about these nicotine products.

## Figures and Tables

**Table 1:** Study Participants’ Demographics, N = 679.

	All	E-Cigarettes		χ^2^ (*p*-Value)
		Non-Users	Ever Users	
		427 (62.9)	252 (37.1)	
Age				(0.579) ^&^
Mean ± SD	20.5 ± 2.1	20.5 ± 2	20.5 ± 2.1	
Median (IQR)	20 (2)	20 (2)	20 (2)	
Gender				44.8 (0.000)
Male	153 (22.5)	61 (39.9)	92 (60.1)	
Female	526 (77.5)	366 (69.6)	160 (30.4)	
Nationality				0.6 (0.543)
Jordanian	629 (92.6)	393 (62.5)	236 (37.5)	
Other	50 (7.4)	34 (68)	16 (32)	
School/Specialty				24.3 (0.000)
School of Medicine	108 (15.9)	68 (63)	40 (37)	
School of Dentistry	140 (20.6)	80 (57.1)	60 (42.9)	
School of Pharmacy	150 (22.1)	114 (76)	36 (24)	
School of Rehabilitation Sciences	167 (24.6)	86 (51.5)	81 (48.5)	
School of Nursing	114 (16.8)	79 (69.3)	35 (30.7)	
Academic Level				8.7 (0.019)
First	143 (21.1)	85 (59.4)	58 (40.6)	
Second	155 (22.8)	92 (59.4)	63 (40.6)	
Third	128 (18.9)	78 (60.9)	50 (39.1)	
Fourth	157 (23.1)	99 (63.1)	58 (36.9)	
Fifth and higher	96 (14.1)	73 (76)	23 (24)	
GPA				17.5 (0.002)
Excellent	140 (20.6)	99 (70.7)	41 (29.3)	
Very good	322 (47.4)	212 (65.8)	110 (34.2)	
Good	159 (23.4)	91 (57.2)	68 (42.8)	
Fair	49 (7.2)	20 (40.8)	29 (59.2)	
Weak	9 (1.3)	5 (55.6)	4 (44.4)	
Place of Residence				3.1 (0.087)
Amman	499 (73.5)	304 (60.9)	195 (39.1)	
Others	180 (26.5)	123 (68.3)	57 (31.7)	
Living condition				8.5 (0.014)
With family	607 (89.4)	393 (64.7)	214 (35.3)	
With friends	14 (2.1)	7 (50)	7 (50)	
Alone	58 (8.5)	27 (46.6)	31 (53.4)	
House				0.9 (0.373)
Owned	542 (79.8)	336 (62)	206 (38)	
Rented	137 (20.2)	91 (66.4)	46 (33.6)	
Monthly Allowance				15.7 (0.000)
Less than 100 JD ($140)	333 (49)	234 (70.3)	99 (29.7)	
100 JD ($140)–250 JD ($350)	268 (39.5)	152 (56.7)	116 (43.3)	
More than 250 JD ($350)	78 (11.5)	41 (52.6)	37 (47.4)	

SD: Standard Deviation; IQR: Inter Quartile Range; GPA: Grade Point Average; ^&^ Mann Whitney test; Data is presented as N (%) unless otherwise mentioned.

**Table 2:** Study Participants’ Exposure to E-cigarettes, N = 679.

	All	E-Cigarettes		χ^2^ (*p*-Value)
		Non-Users	Ever Users	
Father’s Education				4.3 (0.045)
School degree or less	199 (29.3)	137 (68.8)	62 (31.2)	
Diploma or more	480 (70.7)	290 (60.4)	190 (39.6)	
Mother’s Education				0.9 (0.382)
School degree or less	198 (29.2)	130 (65.7)	68 (34.3)	
Diploma or more	481 (70.8)	297 (61.7)	184 (38.3)	
Conventional cigarette user				100 (0.000)
Yes	39 (5.7)	1 (2.6)	38 (97.4)	
No	619 (91.2)	425 (68.7)	194 (31.3)	
Ex-smoker	21 (3.1)	1 (4.8)	20 (95.2)	
Father ever used e-cigarettes				11.5 (0.003)
Yes	199 (29.3)	106 (53.3)	93 (46.7)	
No	457 (67.3)	307 (67.2)	150 (32.8)	
I don’t know	23 (3.4)	14 (60.9)	9 (39.1)	
Mother ever used e-cigarettes				35.9 (0.000)
Yes	78 (11.5)	26 (33.3)	52 (66.7)	
No	593 (87.3)	398 (67.1)	195 (32.9)	
I don’t know	8 (1.2)	3 (37.5)	5 (62.5)	
Siblings ever used e-cigarettes				94.2 (0.000)
Yes	338 (49.8)	152 (45)	186 (55)	
No	311 (45.8)	254 (81.7)	57 (18.3)	
I don’t know	30 (4.4)	21 (70)	9 (30)	
Friends ever used e-cigarettes				142.9 (0.000)
Yes	464 (68.3)	222 (47.8)	242 (52.2)	
No	167 (24.6)	162 (97)	5 (3)	
I don’t know	48 (7.1)	43 (89.6)	5 (10.4)	

Data is presented as N (%).

**Table 3:** Frequency and Duration of E-cigarette Use and Source of Information about E-cigarettes as Reported by Study Sample, N = 679.

	All	E-Cigarettes		
		Non-Users	Ever Users	
The duration of e-cigarette use				
I have just tried it	118 (17.4)	NA	118 (46.8)	NA
Less than 1 year	60 (8.8)	NA	60 (23.8)	NA
1–3 years	60 (8.8)	NA	60 (23.8)	NA
More than 3 years	14 (2)	NA	14 (5.6)	NA
Frequency of e-cigarette use				
I have just tried it	118 (17.4)	NA	118 (47.8)	NA
Daily	72 (10.6)	NA	72 (28.6)	NA
Weekly	23 (3.4)	NA	23 (9.1)	NA
Monthly	39 (5.6)	NA	37 (15.5)	NA
Source of information about e-cigarettes				
Media (TV, radio … etc.)	332 (48.9)	240 (72.3)	92 (27.7)	24.6 (0.000)
Social media	549 (80.9)	356 (64.8)	193 (35.2)	4.7 (0.020)
Friends	541 (79.7)	301 (55.6)	240 (44.4)	59.9 (0.000)
University lectures and seminars	394 (58)	274 (69.5)	120 (30.5)	17.8 (0.000)
Scholarly journal (Research articles)	202 (29.7)	140 (69.3)	62 (30.7)	5.1 (0.024)
Society	25 (3.7)	19 (76)	6 (24)	1.9 (0.208)
Family	35 (5.2)	24 (68.6)	11 (31.4)	0.5 (0.591)
Tobacco shops	8 (1.2)	4 (50)	4 (50)	0.6 (0.478)

Data is presented as N (%).

**Table 4:** Study Participants’ Level of Knowledge about E-cigarettes, N = 679.

Item/Measure	All	E-Cigarettes		*p*-Value
		Non-Users	Ever Users	
E-cigarettes contain nicotine.	491 (72.3)	308 (72.1)	183 (72.6)	0.929 ^$^
E-cigarettes are considered carcinogenic.	491 (72.3)	282 (66)	209 (82.9)	0.000 ^$^
E-cigarettes are considered a tobacco product.	480 (70.7)	310 (72.6)	170 (67.5)	0.163 ^$^
E-cigarettes cause addiction/are addictive.	350 (51.5)	214 (50.1)	136 (54)	0.341 ^$^
E-cigarettes are an FDA approved product.	73 (10.8)	31 (7.3)	42 (16.7)	0.000 ^$^
Total Knowledge Score, Mean ± SD	2.8 ± 1.1	2.7 ± 1.2	2.9 ± 1	0.019 ^&^

Data are presented as N (%) of those who correctly answer the question; the percentage of students who answer correctly in each group per item are shown; ^$^ Chi-square test; ^&^ Mann Whitney test.

**Table 5:** Study Participants’ Attitude toward E-cigarettes, N = 679.

	All	E-Cigarettes		
		Non-Users	Ever Users	*p*-Value *
	Mean ± SD	Mean ± SD	Mean ± SD	
Accessibility	5.8 ± 1.6	6.1 ± 1.6	5.3 ± 1.5	0.000
Acceptability	6 ± 1.3	5.9 ± 1.3	6.1 ± 1.4	0.030
Safety	12.2 ± 2.1	11.9 ± 2	12.8 ± 2.1	0.000
Supervision	9 ± 2.8	8.7 ± 2.7	9.6 ± 3	0.000
Restriction	6.2 ± 1.9	6 ± 1.8	6.5 ± 2	0.000

* Independent sample *U*-Mann Whitney test.

**Table 6:** Covariates Affecting Students from Health Professions Schools Being E-cigarettes Ever Users Using Logistic Regression Model.

	OR (95% CI)	*p*-Value
Gender being male	2.3 (1.4–3.8)	0.002
Mother ever used e-cigarettes	2.4 (1.3–4.4)	0.003
Siblings ever used e-cigarettes	3.3 (2.3–4.9)	0.000
Friends ever used e-cigarettes	4.4 (2.4–7.9)	0.000
Source of information about e-cigarettes is media	1.6 (1–2.6)	0.041
Accessibility score	0.7 (0.6–0.8)	0.000
Safety score	1.1 (1–1.2)	0.037

OR: Odds Ratio.

## Data Availability

The data presented in this study are available on request from the corresponding author. The data are not publicly available to assure confidentiality.
